# Integration of Deworming into HIV Care and Treatment: A Neglected Opportunity

**DOI:** 10.1371/journal.pntd.0001738

**Published:** 2012-07-31

**Authors:** Helen L. Gerns, Laura R. Sangaré, Judd L. Walson

**Affiliations:** 1 Department of Epidemiology, University of Washington, Seattle, Washington, United States of America; 2 Department of Global Health, University of Washington, Seattle, Washington, United States of America; 3 Department of Medicine, University of Washington, Seattle, Washington, United States of America; London School of Hygiene & Tropical Medicine, United Kingdom

In sub-Saharan Africa, where two-thirds of all HIV-infected individuals reside, many are now aware of their HIV infection status and millions are receiving antiretroviral therapy. In many countries in the region, new infections are declining and individuals are living longer with treatment to control their HIV infection. The infrastructure behind HIV care and treatment is vital in addressing the HIV pandemic, and similarly could be utilized to address and promote other health issues specific to persons living with HIV.

An estimated 2.1 million children are infected with HIV in sub-Saharan Africa, where multiple helminth species are also endemic [1]. Likely half of these children are co-infected with helminths [2]. The most recognized consequences of helminth infection in children include anemia, malnutrition, and impaired cognitive development, which are independent risk factors for death among HIV-infected children. Standard treatment of soil-transmitted helminth infection entails a single 400 mg dose of albendazole [3], making routine deworming of children a simple intervention that safely and affordably prevents the adverse effects of chronic helminth infection. Deworming HIV-infected children, specifically, may have a substantial impact on child health through the synergistic effects of improved nutritional status, greater control of other infectious diseases, and increased vaccine responsiveness, and therefore should be provided though HIV care services [3].

Helminth infection in HIV-infected children may impact how the host responds to infectious diseases and immunizations, indirectly through the pathway of malnutrition, as well as directly through immunologic mechanisms. Beyond nutritional deficits, helminths also induce immunosuppressive responses, creating an ideal environment for chronic helminth infection, and inhibiting the host's ability to control other diseases such as HIV [4], [5]. Clinical studies suggest that deworming HIV-infected individuals may delay HIV progression, as measured by CD4 count and HIV viral load [6]–[9]. It is plausible that in addition to improving nutritional status, eliminating helminths in infected individuals may directly impact control of other infectious diseases such as HIV.

Helminth infection may also undermine the benefits of childhood immunizations, through malnutrition, and also by diminishing immune responses to vaccines, both at the time of vaccination and at disease exposure. Population-level data show that regional variations in vaccine efficacy correlate with variations in the prevalence of enteric pathogens [10]. For example, rotavirus vaccine efficacy may be 50% higher in developed countries compared to Africa and Asia [11]. Polio eradication efforts have also been challenged by diminished efficacy of the oral polio vaccine in India as compared to the rest of the world [12]. While the distribution of soil-transmitted helminths represents only one of several factors contributing to these regional variations in vaccine responsiveness, it is a factor which can be easily targeted and controlled through routine deworming [13].

Individual-level evidence also suggests that helminth infection impacts immunologic responses to vaccines. Experimental human and animal studies have shown deworming before immunization increases protective antibody titers, while decreasing immuno-regulatory cytokines [14]–[16]. Children who failed to respond to oral poliovirus vaccination were 25% (*p* = 0.04) more likely to harbor infections with intestinal parasites than vaccine responders [17]. Additionally, children with ascariasis who received albendazole prior to receiving oral cholera vaccine were 88% (*p* = 0.06) more likely to seroconvert than children who were not dewormed [18]. Interactions that diminish responses to vaccines at the time of vaccination may also diminish immune recall of vaccines at the time of disease exposure. As HIV-infected children are more susceptible to vaccine preventable illness and death than other children [19], even after the introduction of anti-retroviral therapy [20], deworming HIV-infected children may have a measurable impact on vaccine preventable infections.

The World Health Organization (WHO) recommends annual or bi-annual school-based deworming as a cost-effective strategy to diminish the consequences of chronic helminth infection. Deworming could also be considered part of the nutritional care package for HIV-infected children, to reduce the consequences of malnutrition and anemia in HIV. Incorporating deworming into routine HIV care and treatment is an ideal way to improve the nutritional health of HIV-infected children, and may provide additional benefits. This may be particularly beneficial for children under 5 years of age, who represent 10%–20% of the 2 billion people infected with helminths worldwide [21]. Annual deworming of preschool-age children is safe and highly effective in reducing parasite prevalence and intensity, malnutrition, and risk of stunting, but a formal policy does not yet exist to target this age group [21], [22]. Because children are infected and often diagnosed with HIV while very young, preschool-aged children can easily be dewormed in HIV clinics, along with siblings, to reduce the occurrence of reinfection.

Despite WHO recommendations, school-based implementation is not universal and many helminth-infected school-age children go untreated. Children who are sick or otherwise unable to attend school may miss school-based interventions, leading to more illness and absenteeism. Children with HIV may also be less likely to receive other health services. For example, HIV-infected children are less likely to receive complete vaccination series compared to uninfected children [23]. HIV care centers are an important, and highly accessed, point of serial contact for HIV-infected children and their families [24]. However, at present they often provide a narrow range of services. In addition to integrating deworming into current HIV treatment, the integration of other necessary childhood health interventions, including vitamin supplementation, immunizations, safe drinking water (through home water filtration), and insecticide-treated bed nets, may further reduce HIV-related morbidity and mortality among these children [24].

Challenges may exist in coupling other health interventions to HIV care, but the potential benefits warrant consideration. Deworming both in schools and HIV clinics is likely justified by the high rates of recurrent infection in children and the low cost of the intervention. Finally, little evidence exists on the impact of deworming in HIV-infected children, highlighting a need for more rigorous studies. These studies should investigate the effects of helminth infection on responses to immunizations, the potential interactions between antihelminthics and HIV treatment, the optimal timing of deworming around both immunizations and ART initiation, and the impact of deworming on incidence of vaccine preventable infections.

The benefits of treating and preventing helminth infections in HIV-infected children may go beyond the improved nutritional status and cognitive development observed in all children to also include improved responses to immunizations and control of other infectious diseases. Enhanced control of neglected infectious diseases, such as helminth infections, through existing HIV care and treatment programs, may further reduce childhood morbidity and mortality in this vulnerable population.
